# Meeting report from the fourth meeting of the Computational Modeling in Biology Network (COMBINE)

**DOI:** 10.4056/sigs.5279417

**Published:** 2014-03-15

**Authors:** Dagmar Waltemath, Frank T. Bergmann, Claudine Chaouiya, Tobias Czauderna, Padraig Gleeson, Carole Goble, Martin Golebiewski, Michael Hucka, Nick Juty, Olga Krebs, Nicolas Le Novère, Huaiyu Mi, Ion I. Moraru, Chris J. Myers, David Nickerson, Brett G. Olivier, Nicolas Rodriguez, Falk Schreiber, Lucian Smith, Fengkai Zhang, Eric Bonnet

**Affiliations:** 1 Department of Systems Biology and Bioinformatics, University of Rostock, Rostock, Germany; 2 Department of Computing and Mathematical Sciences, California Institute of Technology, Pasadena, California, USA; 3 IPK Gatersleben, Gatersleben, Germany; 4 Instituto Gulbenkian de Ciência - IGC, Rua da Quinta Grande, Oeiras, Portugal; 5 Department of Neuroscience, Physiology and Pharmacology, University College London, United Kingdom; 6 School of Computer Science, The University of Manchester, Manchester, UK; 7 Heidelberg Institute for Theoretical Studies, Heidelberg, Germany; 8 EMBL-European Bioinformatics Institute, Wellcome Trust Genome Campus, Hinxton, Cambridge, United Kingdom; 9 Department of preventive medicine, Keck School of Medicine, University of Southern California, Los Angeles, CA, USA; 10 Center for Cell Analysis and Modeling, University of Connecticut Health Center, Farmington, CT, USA; 11 Department of Electrical and Computer Engineering, University of Utah, USA; 12 Auckland Bioengineering Institute, University of Auckland, Auckland, New Zealand; 13 Systems Bioinformatics, VU University Amsterdam, Amsterdam, The Netherlands; 14 The Babraham Institute, Babraham Research Campus, Cambridge, United Kingdom; 15 Martin Luther University Halle-Wittenberg, Halle, Germany; 16 Monash University, Melbourne, Australia; 17 Computational Biology Unit, Laboratory of Systems Biology, National Institute of Allergy and Infectious Diseases, NIH, Bethesda, Maryland, USA.; 18 Institut Curie, Paris, France; 19 INSERM U900, 75248 Paris, France; 20 Mines ParisTech, 77300 Fontainebleau, France

## Abstract

The Computational Modeling in Biology Network (COMBINE) is an initiative to coordinate the development of community standards and formats in computational systems biology and related fields. This report summarizes the topics and activities of the fourth edition of the annual COMBINE meeting, held in Paris during September 16-20 2013, and attended by a total of 96 people. This edition pioneered a first day devoted to modeling approaches in biology, which attracted a broad audience of scientists thanks to a panel of renowned speakers. During subsequent days, discussions were held on many subjects including the introduction of new features in the various COMBINE standards, new software tools that use the standards, and outreach efforts. Significant emphasis went into work on extensions of the SBML format, and also into community-building. This year’s edition once again demonstrated that the COMBINE community is thriving, and still manages to help coordinate activities between different standards in computational systems biology.

## Introduction

The *Computational Modeling in Biology Network* (COMBINE) [1] coordinates the development of open standards and file formats in computational systems biology and related fields. Recent decades have witnessed a major shift in biological science, with massive amounts of quantitative data increasingly being generated at high speed. Multiple sources of data, such as measurements of the activity and states of various components of cells and tissues, are being integrated into computational models that today help researchers investigate the dynamic properties of living systems. Various online resources disseminate curated information related to pathways (e.g., WikiPathways [2], PANTHER pathways [3], KEGG [4], Reactome [5], Pathway Interaction Database [6]) and computational models (e.g., BioModels Database [7], Physiome Model Repository [8]). The growth of these resources has led to the creation of open standards to facilitate the exchange and interoperability of models and data, as well as the development of computational software tools. The Systems Biology Markup Language (SBML) [9] covers computational models of biological processes, describing variables, their relationships and initial conditions. BioPAX, the Biological Pathway eXchange format [10], focuses on the representation of biological pathways at the molecular and cellular levels. The Systems Biology Graphical Notation (SBGN) [11] is a set of visual languages enabling the graphical representation of biological processes. Additional standardization efforts under the COMBINE umbrella include CellML [12], aimed at storing and exchanging computer-based mathematical models; NeuroML [13], a language for the description of detailed models of neural systems; and the Simulation Experiment Description Markup Language (SED-ML) [14], an XML-based format for encoding the configurations and procedures necessary to reproduce computational experiments on models. All efforts use a common development approach in which the community is the central driver: community participation is actively pursued not only to identify and suggest novel features to include in the standards, but also to choose the editorial board members and make other technical decisions democratically. Finally, the COMBINE standards incorporate a number of related resources, including ontologies such as the Systems Biology Ontology (SBO) [15], and Minimum Information Guidelines such as MIRIAM [16], which are again actively being evolved by the community.

Unfortunately, many of these standards were originally developed independently, with poor coordination and consequently little or no integration between the different efforts. As a result, redundancies in topic coverage and efforts arose, wasting scarce sources of funding and time. The COMBINE initiative was created to help avoid these obstructions to smooth progress. It was inspired by the way in which the World Wide Web Consortium (W3C [17]) develops standards for the web. COMBINE has succeeded in creating a number of community events aimed at coordinating and promoting the development of open and interoperable standards for computational systems biology [18].

The *COMBINE Forum* is an open event that offers the opportunity to discuss new developments and features in the different standards, address cross-standard interoperability issues, and to hear about implementation and scientific work that benefits (or can benefit) from the use of the standards. This meeting is attended predominantly by developers of (proposed) standards, software developers who implement support for these standards, and interested end users of COMBINE standards. The COMBINE Forum was launched three years ago and has since has been accepted as *the* cross-standard community meeting. It first took place in 2010, organized by the center for integrative systems biology at the University of Edinburgh, United Kingdom, as a joint event with the 10th SBML Anniversary and a satellite of the 11^th^ ICSB [19]. Since then, the meetings have taken place in Heidelberg (2011, organized by the *Heidelberg* Institute for Theoretical Studies, Germany) and in Toronto (2012, organized by the Donnelly Centre in Toronto, Canada).

The second type of annual meeting organized by the COMBINE initiative is HARMONY, the *Hackathon on Resources for Modeling in Biology*. As the name indicates, it is organized as a hackathon and is targeted towards software developers, favoring hands-on sessions instead of presentations.

A number of outreach activities have been implemented throughout the past several years to promote open computational standards for biology. Joint tutorials took place as 1-day satellite workshops of the International Conferences on Systems Biology (ICSB) 2012 in Toronto, Canada [20] and 2013 in Copenhagen, Denmark [21]. At the core of these workshops, an international team of tutors introduced the most widely used computer tools, model databases and data management platforms in the field of systems biology and provided instructions in how these tools support COMBINE standards. Both tutorials were well attended with 80-100 participants.

**Figure 1:**
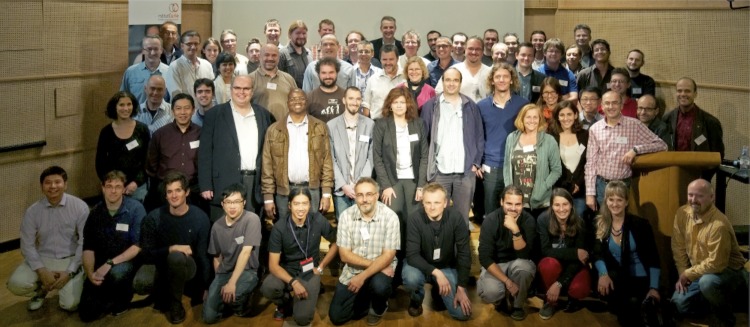
Group picture of the participants to COMBINE 2013 (photo courtesy of Mike Hucka).

In September 2013, the members of the community gathered in Paris for their annual forum meeting (Fig. 1). The COMBINE Forum was hosted by the *Computational Systems Biology of Cancer* group of the Institut Curie [22]. The organizers devoted the first day of the meeting to general presentations on modeling approaches in biology. The focus was on approaches that either are not yet covered by COMBINE efforts, or that are being used in new or innovative ways. No fee was charged for attending this first day and the audience reached 96, including many students. Days 2 through 5 were then dedicated to recent developments in COMBINE standards, and discussion on future evolutions. As with previous COMBINE events, slides and video recordings of all presentations are available on the meeting website [23].

## Modeling approaches in biology (Day 1)

The COMBINE 2013 scientific board succeeded in attracting an impressive panel of speakers for the first day of the meeting. The speakers represented various and exciting areas of modeling applied to biology (see Table 1 for the schedule).

**Table 1:** Schedule Day 1 of the COMBINE 2013 meeting

**Session 1**	**Chair: Andrei Zinovyev**
Michael Hucka, Caltech	Recent developments in the world of SBML (the Systems Biology Markup Language)
Frank T. Bergmann, Heidelberg University	Applying the Scientific Method to Simulation Experiments: SED-ML
Vincent Danos, CNRS, University Paris-Diderot, University of Edinburgh	Rule-based approaches to modeling
Denis Thieffry, École Normale Supérieure	Logical modeling of cell fate specification
**Session 2**	**Chair: Emmanuel Barillot**
Marco Antoniotti, BIMIB	Modeling Colonic Crypts with VCell and SBML/Spatial
Hiroaki Kitano, SBI, OIST	Garuda Platform: An integrated inter-operability for biomedical software and data resources
Andrew Davison, CNRS	Interoperability and model sharing in large-scale neuronal network modeling and neuromorphic computing
Marc Lavielle, INRIA, University Paris-Sud	Monolix and other new tools for population pharmacometrics
**Session 3**	**Chair: Eric Bonnet**
Hervé Turlier, Institut Curie	A model for furrow constriction in animal cell cytokinesis
Benjamin Ribba, INRIA	Modeling of efficacy data in clinical oncology
Andrei Zinovyev, Institut Curie	Mathematical modeling of cancer-related molecular mechanisms
Dirk Drasdo, INRIA, University of Leipzig	Agent-based models of tissue organization - concepts and components

The meeting was opened by Eric Bonnet (Institut Curie, France), the local organizer, who welcomed everyone and thanked the sponsors of the meeting. Nicolas Le Novère (Babraham Institute, United Kingdom) then informed the participants about the latest updates in the COMBINE network and about the organization and purpose of this year’s meeting.

The first invited speaker of the day was Michael Hucka (California Institute of Technology, United States) who gave an overview of SBML, and summarized recent developments of the language. Of particular interest are two new additions to the SBML Test Suite [112]: a graphical standalone desktop program that allows users to run an SBML-compatible application through a series of test cases, and an online database of SBML Test Suite results. Dr. Hucka also described how the SBML specification is still growing, and now includes many so-called *packages* that add constructs on top of SBML Level 3 Core, such as the ability to compose models hierarchically (using the *Hierarchical Model Composition* package), represent constraint-based models in a tool-independent format (using the *Flux Balance Constraints* package), and represent Petri net and logical models (using the *Qualitative Models* package). The next speaker was Frank T. Bergmann (California Institute of Technology, United States, and University of Heidelberg, Germany) who described the rationale behind the development of SED-ML [14], a markup language designed for encoding simulation setups, and ensuring the exchangeability and reproducibility of experiments. Vincent Danos (University Paris-Diderot, France, and University of Edinburgh, United Kingdom) then presented an approach to modeling biological processes that involves rule-based descriptions of mechanisms and phenomena. His work is embodied in the Kappa language ([24], [113]). When compared to a number of other approaches, rule-based systems demonstrate certain advantages, such as compact descriptions, an intrinsic ability to handle the combinatorial nature of biological interactions, and a better mapping between the structures of data source and model. Dr Danos also presented a novel approach still in development: energy-based modeling. Denis Thieffry (Institut de Biologie de l’Ecole Normale Supérieure, France) gave the final presentation of the morning session. He began his talk with a general overview of the various flavors of logical modeling. Logical modeling has several important advantages, such as the ability to exploit incomplete, heterogeneous and qualitative sources of data, a rigorous formal framework and a straightforward way to simulate perturbations. Dr. Thieffry is coordinating the development of the logical modeling tool GINsim [25], and is also involved in the development of the SBML *Qualitative Models* package [26]. The last part of his presentation was show cased how logical modeling can be applied to study the differentiation of T-helper cells [27].

After a quick lunch, the first speaker in the afternoon was Marco Antoniotti (University of Milano Bicocca, Italy), with a talk selected from the abstracts submitted to the meeting. Dr. Antoniotti described his investigation into modeling intestinal crypts using the SBML *Spatial Processes* package and the Virtual Cell software environment [28]. He discussed several issues he faced, such as the insufficient interplay between static and dynamic representations, inconsistencies between the SBML *Spatial Processes* package and other SBML packages, and the difficulty of replicating spatial simulations. The next speaker was Hiroaki Kitano (Systems Biology Institute and Okinawa Institute of Science and Technology, Japan), one of the founders of Systems Biology and the initiator of the projects that gave rise to SBML and SBGN. Professor Kitano presented PhysioDesigner [29,114], an open software platform supporting multilevel modeling for physiological systems. The platform integrates the formats SBML, CellML and PHML, a language expressing hierarchies and the dynamics of biophysical functions. PhysioDesigner is compatible with the Garuda project, an ongoing effort to develop a computational and knowledge platform for healthcare research that can be used in both academic and industrial environments. Andrew Davison (CNRS, France) then presented the problems linked to large-scale neuronal networks modeling, as well as some software tools for collaborative modeling, model sharing with tools, and formats such as PyNN [30,31] and NeuroML. Marc Lavielle (INRIA, France) introduced population pharmacometrics to the audience, presenting models, methods and tools linked to this field. He notably introduced the modeling language MLXTran and the platform MLXPlore [32]. MLXPlore is a graphical software system for the exploration and visualization of pharmacometrics models. MLXTran models can be run from MATLAB or R. Right after the coffee break, the last session of the day began with a short presentation from Hervé Turlier, a PhD student at the Institut Curie, on his biomechanical model of cell cytokinesis. Benjamin Ribba (INRIA Grenoble, France) then showed his work on modeling efficacy data in clinical oncology. More often than not, only a few measurements are performed to determine the efficacy of a given therapeutic treatment. In his presentation, Dr. Ribba showed a case study where clinical data was used to efficiently model the dynamic response of a tumor to antitumor treatment [33]. Andrei Zinovyev (Institut Curie, France) then presented the latest developments of BiNoM, a software tool for the analysis of large-scale molecular maps encoded in open standard formats [34]. Dr. Zinovyev went on to detail a case study in the use of logical modeling of cell-fate decision in response to death receptor engagement [35]. The last speaker of the day was Dirk Drasdo (INRIA Rocquencourt, France, and University of Leipzig, Germany). Dr. Drasdo made the case that multicellular systems are inherently multiscale in character. Agent-based modeling is a natural way of approaching this type of problem and has been used extensively in biology [36]. Dr. Drasdo presented an application of agent-based modeling to the study of liver cell regeneration after damage in mice, a modeling approach that should be broadly applicable for systems biology of tissues [37].

In summary, Day 1 featured high-profile scientific presentations about state-of-the-art modeling activities and highlighted how these might incorporate standard formats. Interestingly, the talks were attended by people with very different backgrounds, offering novel possibilities for modelers to exchange experiences in working with standards, and to get in contact with the standards developers.

## The core COMBINE Forum (Days 2-5)

The remaining four days of the meeting focused on the various COMBINE standards, recent development and future work. Major topics of discussion were SBML developments, the linking of models and data, metadata standards, and the distribution of models through open repositories (see Table 2 for the schedule). Discussions about the different SBML packages took place during all days of the meeting.

**Table 2:** Schedule Days 2-5 of the COMBINE 2013 meeting.

**Tue Sep 17**	**Wed Sep 18**	**Thu Sep 19**	**Fri Sep 20**
Update on SBML, CellML, NeuroML and PharmML (chair: Nicolas Le Novère)	Data representation and use (chair: Frank T. Bergmann)	Model sharing (1): Metadata (chairs: Nick Juty and Dagmar Waltemath)	SBGN: overview and update (chair: Falk Schreiber)
SBML Level 3 Version 2 (chair: Mike Hucka)	SED-ML future: L2 / L1V3, parameter estimation (chair: David Nickerson)	Model sharing (2): Repositories (chairs: Nick Juty and Dagmar Waltemath)	Visual Markup (SBGN-ML, SBML Layout etc.) (chair: Tobias Czauderna)
Using distributions in model descriptions (chair: Lucian Smith) SBML Flux Balance Constraints (chair:Brett Olivier)	SBML Spatial (chair: Ion Moraru)	COMBINE archive (chair: Nicolas Rodriguez) PALS meeting (chairs: Olga Krebs, Carole Goble)	SBML Arrays & Hierarchical Model Composition Packages (chair:Lucian Smith) SBGN: development of L2 (chair: Huaiyu Mi) PALS meeting
SBML Qualitative Modeling package (chair: Claudine Chaouiya) modeling physiology (chair: Padraig Gleeson)	Community building and links with other efforts (chair: Martin Golebiewski)	SBML Multi package (chair: Fengkai Zhang) BioPAX TC PALS meeting	SBML Dynamics Package (chair:Chris Myers) SBGN: development of L2 (chair: Falk Schreiber) PALS meeting

## COMBINE standards and SBML Packages (Day 2, Day 3 and Day 5).

### Update on SBML, CellML, NeuroML and PharmML

David Nickerson (Auckland Bioengineering Institute, New Zealand) started day two of the meeting with an introduction to the current state of the CellML project. He gave some recent examples of how CellML is being used in large-scale physiological models. These examples covered a range of spatial scales (single cell to whole heart) and types of physical processes (fluid dynamics, mechanical deformation, and electrical propagation). Dr. Nickerson also highlighted the computational demands such large scale models place on numerical simulations and presented some work being done in the CellML community to address them, including software-based optimizations and hardware acceleration. The use of GitHub for the future development of the CellML specification [38] was also briefly discussed, as were the changes being introduced in the next version of the CellML specification which is currently being drafted by the CellML editorial board. A summary of the main CellML-capable tools currently available highlighted the main benefits and capabilities of each tool. The new OpenCOR [39] tool was presented as a recent development of potential interest to the COMBINE community.

Padraig Gleeson (University College London, United Kingdom) then gave an update on developments towards NeuroML version 2.0 [40]. This new version is in active development and differs from the previous by having the structural and dynamical behavior of the language elements (cells, ion channels, synapses, etc.) specified in a machine-readable format as opposed to described in human-readable text [13]. This underlying language, LEMS (Low Entropy Model Specification), facilitates mapping of NeuroML elements to other formats, including a range of neuronal simulators such as NEURON [41] and BRIAN [42], and also enables interoperability with other model representation formats such as SBML. An increasing number of NeuroML models are being made available on the Open Source Brain repository for collaborative model development in computational neuroscience [43]. Formal specifications for NeuroML 2 and LEMS are being developed by the recently elected NeuroML Editorial Board.

Maciej Swat (EMBL-EBI, United Kingdom) closed the session by presenting the progresses of the Pharmacometics Markup Language, PharmML, a new format to describe pharmacometrics models and clinical trials [44]. PharmML is being developed by the Drug Disease Model Resources (DDMoRe [45]), a project funded by the Innovative Medicine Initiatives. PharmML encodes the model definition, trial design and modeling steps. The model definition encompasses more layers than in systems biology, including the structural model (the equation describing the links between variable and their evolution), the covariate model (transformation of a covariate which typically involves scaling or normalizing), the parameter model (describing the distributions of structural model parameters and their relationship with the covariates and variability structure) and the observation model (for instance residual errors).

### SBML Level 3 Version 2

 After a break, Mike Hucka discussed recent developments in SBML. The forthcoming SBML Level 2 Version 5 will correct known issues in SBML Level 2 and introduce a few updates, for instance on resources named in the specification document. SBML Level 3 Version 2 will also include corrections and some updates, including changes designed to facilitate development of SBML Level 3 *packages*. Dr. Hucka also discussed the current state of different SBML Level 3 packages, with special emphasis on three packages that are nearing finalization: the *Multistate and Multicomponent Species* package (SBML *Multi*), which aims to add support for the exchange of rule-based models of elements with different states; the *Spatial Processes* package, which aims to add support for the exchange of models with spatially-distributed entities; and the *Distributions* package, which aims to add support for models with statistical distributions of numerical values.

Dr. Lucian Smith (California Institute of Technology, United States) then spoke more specifically about the proposed changes to be incorporated in SBML Level 3 Version 2. These include lifting restrictions on sub-elements in SBML, potentially allowing packages to provide their own child elements with a replacement meaning. Other planned changes are to add identifiers to elements that currently lack them and lifting restrictions on some types of identifier references, so that identifiers defined by package constructs could be used in the same fashion as core identifiers. Other proposals were more technical, generated good discussions, and resulted in possible avenues for further development of the SBML specification.

### SBML Level 3 Packages

Level 3 packages were discussed in more detail throughout the whole meeting. One session dealt with the use of distributions of numerical values in models, with Maciej Swat and Pedro Mendes (University of Manchester, United Kingdom) presenting information about how their software tools used such distributions. They were followed by a discussion led by Lucian Smith on the proposed SBML *Distributions* package. The software tool discussion provided an excellent anchor for the subsequent SBML discussion, as it allowed participants to connect what the package would contain with what modelers are using in their models. Overall, the dual approach of using the package to allow users to define distribution functions to be used as part of the model, together with descriptions of mathematical elements in terms of distributions and summary statistics, was deemed sufficient to cover what modelers will need. More specifically, UncertML [46] was agreed to be sufficient to describe the necessary distributions.

An enthusiastic group of attendees from a diverse background (developers and modelers alike) attended the session on the SBML *Flux Balance Constraints* package (nicknamed the *FBC* package). Version 1 of the official FBC specification, which was released earlier this year [47], extends SBML by adding support for steady-state constraint based models. This type of model is used, for example, in Flux Balance Analysis (FBA). The session was opened by Brett G. Olivier (VU University Amsterdam, The Netherlands), who gave an overview of the *FBC* package and reference tools (e.g. the FBA Tool [48], and CBMPy/FAME [49,50]). Frank T. Bergmann then described a comprehensive set of tools to complement the libSBML implementation of FBC, including converter to and from the widely-used SBML annotations originated by the COBRA Toolbox [51] the SBML online validator [52]; SBML Test Suite [53], as well as the FBC MATLAB implementation [54]. Finally, Matthias König (Charité, Germany) discussed the latest versions of his tools CyFluxVis [55] and CySBML [56]. Both tools visualize and annotate FBA and kinetic models [56]. The remaining time was spent discussing two major concepts for the next version of the package: GeneAssociations and GenericAnnotation. Gene-protein interactions are commonly found in most existing FBA models; however, there is currently no standard for encoding them. Different proposals had been made and a consensus found at the previous HARMONY had been approved also by this year’s participants. Subsequently, the problems with encoding FBC-specific generic annotations was discussed, with a proposal for a new class of “additional properties” considered and presented to the wider FBC community for comment.

Another session was devoted to the SBML *Qualitative Modeling* package specification that was finalized earlier in 2013 to support the representation of qualitative models [26]. David Cohen (Institut Curie, France) started the session with a presentation of a mathematical model of synthetic dosage gene interactions leading to EMT-like phenotype in vivo. Complementing Denis Thieffry’s talk on the first day, this work illustrated the use of the logical formalism. Then, Aurélien Naldi (UNIL-CIG, Switzerland), the main developer of the software tool GINsim [25], presented LogicalModel, a Java library for manipulating and converting qualitative models. This work is part of the Common Logical modeling Tools initiative [57], a collaboration between numerous groups who are developing and using logical modeling software tools. The session ended with a discussion of future directions, which mainly focused on simulation descriptions and the comparison of results. This topic was also discussed in the SED-ML session on the third day of the COMBINE meeting. A result of the discussions was the decision to start by defining appropriate terms in KISAO, the Kinetic Simulation Algorithm Ontology [15]. Finally, the meeting participants raised a few questions that will need to be addressed in the future, such as the consideration of quantitative values (e.g., delays or probabilities) in qualitative models, as well as the precise nature of interactions between constructs specific to the *Qualitative Models* package and SBML *Core* constructs.

The SBML *Multi* package was discussed in another session. It provides support for models with molecular complexes that have multiple components and can exist in multiple states and in multiple compartments. One goal is to provide a platform for sharing models based on the specifications of bi-molecular interactions and the rules governing such interactions [58-61]. Fengkai Zhang (National Institute of Allergy and Infectious Diseases, NIH, United States) and Martin Meier-Schellersheim (National Institute of Allergy and Infectious Diseases, NIH, United States) developed the package covering the goals and features described in an initial proposal and specification by Nicolas Le Novère and Anika Oellrich in 2010 [62] and including new features permitting the definition of multicompartment species. Fengkai Zhang gave a presentation of the new release of the package specification [62] that permits defining multiple occurrences of *SpeciesFeatureType*, multiple copies of *SpeciesTypeInstance* and a *numericValue* for *PossibleSpeciesFeatureValue,* which enable the package to naturally encode models with identical species components and/or species states and models with arithmetic operations on the numeric values of species states. In addition to the meeting participants, researchers and tool developers interested in the SBML *Multi* package attended the session remotely via *Google Hangouts*. The session concluded with a discussion of potential additions and clarifications. There was agreement that the development of libSBML plug-ins for the SBML *Multi* package could start based on the current specification.

In the afternoon of Day 3, a session was devoted to the SBML *Spatial Processes* package. While the focus was primarily on the SBML package, it also involved presentations and discussions of other standards related to spatial modeling, their respective strength and limitations, and possibilities for interoperability. First, Jim Schaff (University of Connecticut Health Center, USA) presented the current status of the SBML *Spatial* package. An initial draft of the complete specification was released in July 2013, and experimental libSBML binaries are now available, as well as prototype implementations of support for SBML *Spatial* in VCell 5.3 [63], RoadRunner [64,111], and CellDesigner [65]. The first successful exchange of two spatial models between different tools using the *Spatial Processes* package was reported. The current scope of the package is to define the spatial domain for Compartments, to add spatial attributes to Species, Reactions, and Parameters, and to provide geometric definitions for shapes (as of now, supported Geometries are segmented images, inside-outside functions, CSG, polygonal meshes, and signed distance maps). Three presentations on practical use cases followed. Marco Antoniotti built on his presentation from Day 1 on spatial modeling of colorectal crypt dynamics using VCell, this time specifically focusing on the five SBML *Spatial* constructs that were used, and the issues encountered. Fengkai Zhang then presented the Simmune platform [58,60], which uses a rule-based description of molecular interactions and a customized interface for geometrical representation of the spatial simulation context. Finally, David Nickerson presented the spatial representation in CellML by the use of FieldML, and several mixed-standard use-cases. FieldML [66] is a language for representing hierarchical models using generalized mathematical fields. It can be used for exchanging models of 3D physiological structures, descriptions of continuously varying parameters across physical structures and for annotating anatomical models. The possibility of using FieldML to complement SBML *Spatial Processes* was discussed. The general consensus was that the package can now provide comprehensive support for several different representations: reaction/diffusion/advection, particle Brownian dynamics, next subvolume method, and Green functions. This covers at least partially the needs of many current spatial simulation tools, such as VCell [63], JSim [67], Smoldyn [68], MCell [69], E-Cell [70], MesoRD [71]. The most pressing limitations and open issues that were highlighted were: incomplete support for dynamic models, lattice models, initial distributions, and sampled data; as well as the relationship to the SBML *Multi* and *Composition* packages, and to the external standards FieldML and SED-ML.

In the afternoon of Day 5, parallel discussions were held about the SBML *Composition* package, SBML *Arrays* package, and SBML *Dynamic Modeling* package. The session on the *Comp* package focused on software demonstrations of tools implementing support for the package, namely: Antimony [72], BioUML [73], and iBioSim [74]. Discussions resolved a few lingering issues with the Arrays package, and this will enable prototyping of this package to begin. The *Dynamic Modeling* package is still very much under development and while many useful ideas were discussed about the modeling needs, there is still a lot of discussion needed going forward.

### Modeling physiology

 The session on modeling Physiology featured presentations of various initiatives to modeling physiological processes which interact with COMBINE core standards. Randal Britten (New Zealand) presented FieldML via video link from the Auckland Bioengineering Institute. Development of FieldML is closely linked to that of CellML through the Physiome project. Ilya Kiselev (Institute of Systems Biology, Russia) presented BioUML [75], a simulation environment that can be used online or installed locally. BioUML provides many features including integration with biological databases, visualization and scripting support. Martin Golebiewski (Heidelberg Institute for Theoretical Studies, Germany) closed the day with his presentation of how the Virtual Liver network [76] attempts to integrate data across different biological scales. The focus of his talk was on the application of standards in data management within distributed research networks. The first successful example was the seamless integration of data and models in the SEEK platform [77]. The second one was the integration of experimental reaction kinetics data into models through the SABIO-RK database [78,79].

## Data representation, simulation, and community building (Day 3)

### Data representation and simulation

Day 3 of COMBINE 2013 started with a session on how data access can be provided within simulation setups. Frank T. Bergmann summarized previous discussions that took place in meetings relating to SED-ML [14,80] and in between the different working groups over the last year. He highlighted the importance of linking data to simulation experiment descriptions inside SED-ML. The lively discussion led to the conclusion that several core SED-ML components need to be given access to experimental data (the SED-ML Model, the SED-ML Task and the SED-ML DataGenerator). This extension of the current standard for simulation description would in the future enable tools to (1) encode parameterization of models from experimental data, (2) to take advantage of experimental data when executing simulation experiments and (3) to use experimental data in the post-processing of simulation outcomes. At the previous HARMONY meeting in 2013 it was decided that all experimental data referenced by SED-ML would be represented in NuML, the Numerical Markup Language. Joseph Dada (University of Manchester, United Kingdom) gave in his presentation an update on NuML, explaining how NuML was founded from elements of SBRML, the Systems Biology Results Markup Language [81], in order to facilitate its use by other standardization efforts. NuML provides a flexible structure for encoding numerical information that still is searchable and annotatable. Joseph Dada also talked about libNuML [82], a library for reading, writing and manipulating data in NuML format on all operating systems. Following the approach of libSBML, LibNuML is written in C++ and re-uses the XML parsing layer of libSBML. His talk was followed by a discussion on how to encode data in NuML to illustrate its structure. The final talk of this session was given by Maciej Swat on how data is used in PharmML (see Section 3.a). PharmML already encodes several data sources, particularly in its *Observation* model and its *Trial* design. Initially, this information was saved in a tab-delimited text file that could only be understood with prior knowledge. PharmML now encodes the information in a structured manner. The session continued with a demonstration on how precisely the data is stored in PharmML, and with a comparison to NuML that had been presented before. After these presentations the remaining time was used to establish next steps. The approach discussed at HARMONY on integrating data in SED-ML was accepted so far. The next stage will be prototyping.

The second simulation session focused on which new features may be required in SED-ML. David Nickerson started the session by highlighting the additions to be introduced in the new version of the SED-ML specification (Level 1 Version 2 [83]). The main changes since L1V1 are: the introduction of the concept of nesting simulation tasks to greatly expand the range of simulation experiments that can be encoded in SED-ML; and the ability to parameterize simulation algorithms using the newly available algorithm parameters from KiSAO. Claudine Chaouiya (Instituto Gulbenkian de Ciência, Portugal) then summarized the requirements for describing simulation experiments based on qualitative models, with the models themselves encoded in the SBML *Qualitative Modeling* package. While some work is needed to clarify the requirements of various software tools, the discussion generated by Claudine’s presentation concluded that these requirements could mostly be met by the upcoming SED-ML L1V2 and some additions to KiSAO. Following this discussion, Andreas Dräger presented the Systems Biology Simulation Core Library [84]. He stressed the recent addition of support for SED-ML in this software tool and highlighted some issues related to performing simulations based on SBML models as well as the ability of the software to be expanded to support further model encoding standards (e.g., CellML, NeuroML). The session finished with a discussion on other potential features that might be desirable in SED-ML. The purpose of SED-ML was clarified, as a medium for exchanging descriptions of simulation experiments between software tools rather than as a definition of the data structures able to encode all the capabilities of any given software tool. With a large portion of the SED-ML community present at the COMBINE meeting, progress was made turning some of these ideas into feature requests and incorporating them in a future version of the SED-ML specification.

### Community building

The last session of the day was dedicated to the process of community building. The goal of this less technical session was to talk about meta-topics, such as the coordination of the development of the different standards, interoperability, where to publish standards, how to certify standards, how to convince people to use them, etc. Martin Golebiewski started the session with a central question: how should the community interact with other efforts and initiatives in the field? The participants agreed that some of these points have already been successfully addressed. For example, the joint tutorials at the ICSB conferences have been established to train researchers and to promote COMBINE standards. Furthermore, many of the members of COMBINE are also part of larger national and international systems biology initiatives. They promote the usage of the standards within their projects. One conclusion was that we shall continue to organize teaching events that help us establish COMBINE standards as part of the infrastructure backbone in the field of systems biology. Nicolas Rodriguez (EMBL-EBI, United Kingdom) then presented the Systems Biology Format Converter framework (SBFC [85]). SBFC is seen as a fundamental tool for interoperability of COMBINE standards. Another aspect of interoperability is the contact with initiatives that address standardization. As the sizes of systems biology consortia are increasing, standardization of data, formats and workflows is becoming an important foundation for such collaborative efforts. One European network that tries to establish an infrastructure for such distributed research networks is ISBE (Infrastructure for Systems Biology in Europe [86]). This initiative was briefly introduced by Babette Regierer (LifeGlimmer Gmbh, Germany). She stressed that standards can only be established and will only be used throughout the community when they are being promoted in a concerted action that involves all major initiatives in the field.

The last part of the session addressed the question of how specifications of standards could in the future be published to reach a broader audience and be accepted by the systems biology community. Joachim Lonien, innovation manager at DIN (the official German Institute for Standardization), connected this question with the implementation of certification strategies for tools. He gave a brief overview of international standardization organizations and summarized the work of standardization bodies like DIN, the European Committee for Standardization (CEN) or the International Organization for Standardization (ISO). Joachim Lonien also outlined possible ways to support standardization efforts like COMBINE through his organization. More specifically, he suggested that the definition of official standards might help to certify tools that claim to apply these standards. Falk Schreiber (University of Halle-Wittenberg and IPK Gatersleben, Germany) then introduced the idea of “A Unified Publication Platform for Systems Biology Standards” that would involve the cooperation with a scientific journal. He specifically mentioned the Journal of Integrative Bioinformatics [87] as one journal that already offered the publication of standards in a special issue. At the core of this proposal is an annual special issue of a journal containing the current version of each of the standards, as well as current versions of their extensions and packages. Such a special issue would provide freely available and citable specifications of all standards in one place. The additional value for standard developers would be the co-authorship of a journal publication. If and how both suggested approaches to publish COMBINE standards would be compatible remains to be discussed in the future.

## Model repositories and meta-data, COMBINE Archive (Day 4)

### Model repositories and meta-data

 Day four of the meeting focused on model reusability, model management and metadata. Ron Henkel (University of Rostock, Germany) introduced a novel approach for storing models encoded in SBML and CellML and using graph databases [88]. Contrary to existing solutions, his database maps the network structure of models and integrates model-related data such as annotations and simulation descriptions. Ron Henkel also demonstrated how he was able to import all of the path2models data [89] in his database and search it. Another important aspect of model reuse is provenance. Related to this topic, Martin Scharm (University of Rostock, Germany) talked about current issues with version control in model repositories. He introduced an improved method to detect differences in versions of a model [90]. His software tool BudHat [91] lets users compare two versions of an SBML or CellML encoded model, and creates histories for each distinct model. Daniel Arend (IPK Gatersleben, Germany) introduced the e!Dal Data Repository [92,93] as a framework for sharing, versioning and reusing existing data. In particular, he stressed the goal of long-term preservation of data managed inside the e!Dal system, and the importance of metadata when doing so. Dagmar Waltemath (University of Rostock, Germany) concluded the first session with a discussion about a common annotation scheme for all COMBINE standards. Her intention was to homogenize annotations in SBML, CellML, NeuroML, SED-ML (and others) to make these annotated data more comparable on a semantic level.

Tommy Yu (University of Auckland, New Zealand) started the second session with an update on recent developments in the Physiome Model Repository, PMR2 [8,94]. Jacky Snoep (Stellenbosch University, South Africa and University of Manchester, United Kingdom) then presented a case study [95] on how to perform reproducible experiments in JWS Online [96]. Finally, Inna Kuperstein (Institut Curie, France) presented a novel system for the management of cancer-related pathways. The Atlas of Cancer Signaling Networks (ACSN [97] ) is a manually-curated, high-quality collection of signaling pathways known to relate to cancer. These pathways are fully annotated and can be easily navigated to find relevant and rich information [98]. All the molecular maps can be downloaded in BioPAX format and the graphical conventions follow the SBGN standards whenever it is possible.

### Combine Archive

 The focus of the afternoon session was on linking different COMBINE standards. Tobias Czauderna (IPK Gatersleben, Germany) discussed the need for better linking with the example of a generated SBML model and a respective SBGN map: whenever an update of the SBGN occurs, the SBML model should also be updated and vice versa. Similarly, a model update should be reflected in the SED-ML description of the simulation. He summarized several proposals for how cross-linking between COMBINE standards could be realized to overcome these problems. One approach is to use common namespaces, an alternative would be an explicit mapping between entities. The COMBINE archive [99] was introduced by Frank T. Bergman who also presented an implementation supporting the proposed format. Several participants then discussed the COMBINE Archive format and how they plan to implement support for it, for example in PMR2.

## Graphical representations (Day 5)

### SBGN Updates

Day 5 of the meeting focused on the graphical representation of models using SBGN [11]. Falk Schreiber started the session with a summary of the current status of the SBGN effort. He reported on the past SBGN workshop (SBGN-9 [100]) and described two updates to the overall SBGN effort. The first is the creation of a user manual (available from [101]) to help end users learn to read and write SBGN diagrams, while the technical specification would target software and tool developers. The second update was on the progress of SBGN Level 2 development, which was a topic for discussion throughout the day. Stuart Moodie (EBI, United Kingdom) then reported the results of an SBGN survey conducted earlier this year. The purpose of the survey had been to guide the SBGN developers in their next phase of development. From the survey, the community learned that SBGN was used to generate different types of pathway diagrams. The main user base was computational/systems biologists. Most of them expressed concerns about the lack of software support for SBGN, but also responded that it would be high priority to represent pathway knowledge with aesthetically pleasing and unambiguous SBFCs diagrams that could be read without legend, which is the aim of SBGN. Huaiyu Mi (University of Southern California, United States) continued with a presentation about a recent Gene Ontology consortium [102] development trying to capture regulatory relationships among different GO terms annotated to particular gene products. He used the example of the Wnt signaling pathway to show how SBGN-AF could be used in the visual representation of such knowledge. The last two talks of the session were related to the representation of “generics”. Anna Zhukova (INRIA, France) reported her work on using a generalization method to optimally represent large metabolic pathways [101]. Anatoly Sorokin (Institute of Cell Biophysics RAS, Russia) then proposed to use an “identity gate”, corresponding to a generic set representation with containment that captured the relationship between generics and the instances, while maintaining the network connectivity.

### Visual Markup

 The second session focused on markup to encode visual representations. Tobias Czauderna gave an update on the progress of SBGN-ML and LibSBGN development [103] for the next release. He first summarized the current status of the implementation and then outlined features currently being implemented (e.g., the complete support for submaps, and the complete support for (any) compartment shape) and features still under discussion within the community (e.g., support for drawing attributes, and cross-linking between COMBINE standards). Frank T. Bergmann followed with the update on the SBML *Layout* package [1] and on the *Rendering* package [115]. Both specifications had been updated recently by the SBML Level 3 working group. Frank T. Bergmann made a proposition to store color information, or drawing attributes in general, in SBGN-ML. However this feature is still under discussion within the community. The next talk was by Derek Wright (University of Edinburgh, United Kingdom) who presented a software called BioLayout *Express 3D* [104] that converts pathway diagrams in mEPN [105] to SBGN. The session concluded with a few short talks about ideas to reach out and educate bioscience researchers, especially wet-lab scientists. Huaiyu Mi (University of Southern California, United States) presented the idea of a “pathway of the month”, which provides an SBGN redraw of a pathway diagram described in a journal article on the SBGN website. Tobias Czauderna presented Nicolas Le Novère’s idea of a “symbol of the month”, which provides a detailed description of an SBGN symbol each month. He also reported on a recent survey on how SBGN was cited in research papers. The detailed work found that biologists mostly used SBGN-PD to represent signaling pathways in their publications.

The afternoon of Day 5 was mainly dedicated to SBGN Level 2 development. The first part of the session reviewed the meeting notes from the last SBGN workshop (SBGN-9). A detailed summary of discussions related to SBGN Level 2 was given for attendees who had not been present at SBGN-9, and to identify issues requiring clarification or further discussion. The second part of this session dealt with issues related to the usage of SBGN, such as possibilities for simplifying submaps by omitting terminals and tags, and simplifying the composition of hybrid maps by using two or all three SBGN languages in one map, as well as the ease of drawing SBGN maps applied by users omitting particular SBGN glyphs and thereby introducing ambiguity.

## Associated meeting: Satellite SEEK PALs Meeting

The 2013 PALs meeting of the SEEK platform took place as a satellite meeting to COMBINE. The SEEK [106] is a data management and sharing platform which provides an access-controlled, web-based environment for scientists to share and exchange data during day-to-day collaboration and for public dissemination. The “PALs” program was established by the SysMO projects (Systems Biology of Microorganisms [107]) as a regular, direct meeting between the SysMO DB team and co-developers of the platform. This year, the SEEK conducted their PALs meeting as a satellite to the COMBINE meeting to offer to their PALs the opportunity to get more involved with standards and formats in systems biology. The SEEK already follows an incremental, standards-compliant development methodology, encouraging the use of standards, and providing tools for data exploration, annotation and visualization. It furthermore enables linking and management of experiments, protocols, data, models, and publications [108]. Originally developed to address the needs of the SysMO initiative, SEEK has been adopted by over 15 Systems Biology consortia across Europe. The SEEK focus group (also known as the SysMO PALs network) consists of postdocs and PhD students from each of the SysMO projects, covering a broad range of research areas in experimental biology, mathematical modeling and bioinformatics. The PALS are a two way conduit: disseminating developments to their projects/labs and advocating for adoption of the SEEK platform and curation practices, whilst for the developers establishing the real requirements, co-shaping the features needed in SEEK, and testing/validating those features. The PALs team meets several times a year to discuss project requirements and to review new SEEK features. The main themes of this year’s PALs meeting were the curation, annotation and sharing of data and models in order to move SEEK from a consortium resource to a public searchable dissemination resource. The PALs reviewed new SEEK features, discussed integration of Bio ontologies within the data sheets and linking data and models in JWS Online. A detailed agenda of the PALs meeting at COMBINE2013 is available at [109].

The next COMBINE meeting will be organized by the University of Southern California, in L.A., United States [110]. And we hope to see many of you there. If you would like to be kept updated on developments throughout the year, please sign up for the mailing list through our web page.
