# Correction to: Meeting report from the fourth meeting of the Computational Modeling in Biology Network (COMBINE)

**DOI:** 10.1186/s40793-018-0320-4

**Published:** 2018-08-09

**Authors:** Dagmar Waltemath, Frank T. Bergmann, Claudine Chaouiya, Tobias Czauderna, Padraig Gleeson, Carole Goble, Martin Golebiewski, Michael Hucka, Nick Juty, Olga Krebs, Nicolas Le Novère, Huaiyu Mi, Ion I. Moraru, Chris J. Myers, David Nickerson, Brett G. Olivier, Nicolas Rodriguez, Falk Schreiber, Lucian Smith, Fengkai Zhang, Eric Bonnet

**Affiliations:** 1 Department of Systems Biology and Bioinformatics, University of Rostock, Rostock, Germany; 2 Department of Computing and Mathematical Sciences, California Institute of Technology, Pasadena, CA USA; 3 IPK Gatersleben, Gatersleben, Germany; 4 Instituto Gulbenkian de Ciência - IGC, Rua da Quinta Grande, Oeiras, Portugal; 5 Department of Neuroscience, Physiology and Pharmacology, University College London, London, UK; 6 School of Computer Science, The University of Manchester, Manchester, UK; 7 Heidelberg Institute for Theoretical Studies, Heidelberg, Germany; 8 EMBL-European Bioinformatics Institute, Wellcome Trust Genome Campus, Hinxton, Cambridge, UK; 9 Department of preventive medicine, Keck School of Medicine, University of Southern California, Los Angeles, CA USA; 10 Center for Cell Analysis and Modeling, University of Connecticut Health Center, Farmington, CT USA; 11 Department of Electrical and Computer Engineering, University of Utah, Salt Lake City, USA; 12 Auckland Bioengineering Institute, University of Auckland, Auckland, New Zealand; 13 Systems Bioinformatics, VU University Amsterdam, Amsterdam, The Netherlands; 14 The Babraham Institute, Babraham Research Campus, Cambridge, UK; 15 Martin Luther University Halle-Wittenberg, Halle, Germany; 16 Monash University, Melbourne, Australia; 17 Computational Biology Unit, Laboratory of Systems Biology, National Institute of Allergy and Infectious Diseases, NIH, Bethesda, MD USA; 18 Institut Curie, Paris, France; 19 INSERM U900, 75248 Paris, France; 20 Mines ParisTech, 77300 Fontainebleau, Paris, France

## Correction

Following publication of the original article [1], the author requested this addition to the ‘Acknowledgements’ section of the article: “DW is funded by the German Federal Ministry of Education and Research (e: Bio program SEMS, FKZ 031 6194)”.
